# Effects of Geometry and Loading Mode on the Stress State in Asphalt Mixture Cracking Tests

**DOI:** 10.3390/ma15041559

**Published:** 2022-02-18

**Authors:** Yan Li, Weian Xuan, Ali Rahman, Haibo Ding, Bekhzad Yusupov

**Affiliations:** 1 Department of Civil Engineering, Ordos Institute of Technology, Ordos 017000, China; eyyly@oit.edu.cn; 2 Guangxi Key Laboratory of Road Structure and Materials, Guangxi Transportation Science and Technology Group Co., Ltd., Nanning 530029, China; 340113@my.swjtu.edu.cn; 3 School of Civil Engineering, Southwest Jiaotong University, Chengdu 610031, China; haibo.ding@swjtu.edu.cn (H.D.); bekhzad_yusupov@my.swjtu.edu.cn (B.Y.); 4 Highway Engineering Key Laboratory of Sichuan Province, Southwest Jiaotong University, Chengdu 610031, China

**Keywords:** geometry, mechanical load, thermal load, numerical simulation, asphalt mixture, cracking

## Abstract

Currently, a variety of asphalt mixture cracking characterization tests are available as screening tools for the better selection of high-quality raw materials and also for the optimization of mixture design for different applications. However, for a same evaluation index, using different sample geometries and loading modes might lead to obtaining different values, which prevents the application of the evaluation index as a fundamental parameter in pavement design. In this paper, the effects of geometry and loading mode on the stress state in the experimental characterization of asphalt mixture cracking were discussed using numerical simulation. The results showed that applying thermally-induced load in restrained uniaxial test configuration should be considered when performing an asphalt mixture cracking test. Compared with direct tensile configuration, compressive stress clearly existed in other common test configurations, which may prevent the initiation and propagation of cracks. Moreover, it was revealed that nonuniform stress state exists in the dog-bone geometry, which makes it possible to know the failure plane in advance and place gauges at the failure plane for measuring fundamental deformation-related properties.

## 1. Introduction

Asphalt pavement has many advantages compared with concrete pavement, such as installation speed, fast usability, lower maintenance costs, and high skid resistance. In recent years, researchers have also attempted to apply asphalt concrete in trackbed due to its higher vibration attenuation capacity and waterproof function [1]. With the development of construction technology and the optimization of pavement structure, many distresses in asphalt pavement (such as segregation and rutting) can be well controlled. However, premature and excessive cracks in asphalt pavement are still inevitable, especially in cold, northern regions. Environmental temperature, mixture design, asphalt content, and properties of asphalt can all affect the cracking resistance of asphalt mixture. Among these, properties of asphalt is recognized as the most significant factor. Asphalt components have changed significantly over recent decades. On the one hand, industry has continuously increased the dosage of reclaimed asphalt pavement (RAP) for resource utilization efficiency [2]. On the other hand, a variety of waste materials and low-cost additives were used in base asphalt binder to expand its performance grade and reduce its production costs [3,4,5,6,7,8]. All of these practices raised concern about the quality of asphalt binders. Meanwhile, traditional binder characterization methods failed to rule out problematic additives or binder blends owing to the ignorance of the reversible aging phenomenon [9,10,11]. In order to eliminate or retard cracking, some researchers proposed using microcapsules or steel wool to enhance the cracking healing ability of asphalt pavement [12,13]. However, these technologies cannot improve chemically irreversible aging or the reversible aging resistance of asphalt binders. Thus, their long-term effectiveness is doubtful.

Irreversible aging, such as the oxidation of asphalt binders, has attracted increasing attention during recent decades, and several researchers have emphasized its factors. Reversible aging is an isothermal time-dependent hardening phenomenon which may occur at low temperature or medium–ambient temperature, and can be reversed by heating. Low-temperature reversible aging is also called physical hardening, and medium temperature reversible aging is also known as steric hardening. Free volume collapse, asphaltene aggregation, and wax crystallization were identified as the three main factors which lead to the thermal reversible process [14,15,16,17]. Researchers have also developed several empirical and fundamental theoretical models to describe and predict the reversible aging process [18,19,20]. The importance of reversible aging in asphalt mixture is a controversial topic. In the existing literature, few studies on the influence of reversible aging on mixture cracking test results can be found, which can be controlled by stress relaxation in a constrained state [21]. Ontario field pavement trial sections and regular contract testing results showed that low-temperature performance grade of recovered asphalt binder, after going through a physical hardening process, had a better correlation with observed field cracking [22,23]. The most pressing matter of the moment for pavement engineers is to deal with the detrimental influence of reversible aging on asphalt mixture cracking characterization. Only in this way will it be possible to establish a comprehensive theoretical model for asphalt mixture cracking in the future [24]. In addition, before performing a test, it is important to consider the configuration and procedure associated with the test in detail to ensure that the intended test conditions are realized. As the first stage of this research topic, this work was conducted to further investigate the effects of specimen geometry and loading mode on the stress state in asphalt mixture cracking tests through numerical modelling. 

## 2. Methodology

### 2.1. Cracking Characterization of Asphalt Mixture

Generally, an effective approach to prevention of pavement cracking is conducting laboratory experiments to simulate the field failure mode of asphalt mixtures and rank their relative performance. After the implementation of the strategic highway research program (SHRP), a variety of test configurations, parameters, and criterions were proposed to better characterize the cracking resistance of asphalt mixture [25]. Since the main purpose of this study was to compare the effects of existing specimen geometries and loading modes on the stress state in asphalt mixture cracking test methods, the corresponding parameters and evaluation criteria are discussed in detail here. The indirect tensile (IDT) test is one of the most widely used configurations, owing to its simple geometry, low cost of test equipment, and easy implementation [26]. Recently, Zhou, Im, Sun and Scullion [27] proposed a new performance-related cracking parameter from the indirect tensile asphalt cracking test (IDEAL-CT) for asphalt mixture design and quality control (QA) purposes. Low-temperature creep compliance and strength obtained from the IDT test can also be used in the thermal cracking prediction model (TCMODEL) to perform thermal cracking analysis. In order to investigate the variation of pavement properties with depth, Velasquez, Marasteanu, Labuz and Turos [28] proposed a mixture-based bending beam rheology (BBR) test as an alternative test to IDT. For better characterizing the structural properties of asphalt mixture, four-point bending and overlay tensile tests were developed [29]. Moreover, there were some other unnotched testing configurations reported in the literature, such as restrained cooling, dog-bone shape direct tensile, and hollow cylinder tensile tests [30]. The features of unnotched testing configurations are summarized in Table 1. Most of the previous mentioned unnotched tests are based on the linear viscoelastic analysis of creep and strength data. These approaches do not take into account the evolution of cracks with time and are not sensitive to polymer modification type or level [27,28,29,30]; thus, testing on a prenotched sample was proposed. Seven common and representative asphalt mixture fracture characterization methods on notched samples include the single-edge notched beam [31], semi-circular bending [32], the semi-circular tensile [33], the disk-shaped compact tensile [34], the indirect ring tensile [35], the double-edge notched tensile [36], and the restrained notched ring tests [37]. The advantages and disadvantages of these fracture mechanical-based test methods on notched samples are listed in Table 2.

### 2.2. Importance of Thermally-Induced Load on Cracking Behavior of Asphalt Mixture

The main thermal cracking mechanism of asphalt pavement can be described as when the asphalt surface layer is subjected to continuously cooling events, and thermal contraction stress generates within asphalt mixture due to being restrained by the base layer and road shoulder. If the thermal shrinkage stress is higher than the tensile strength of material itself, pavement fracture would be inevitable. Based on this principle, the thermal stress restrained specimen test (TSRST) was developed by Jung and Vinson [25] during SHRP program. However, this test method was not widely used in regular quality assurance (QA) and quality control (QC) procedure owing to the relative complexity and longer time required to apply the thermal cooling in a laboratory. Consequently, many researchers and road agencies apply monotonic mechanical loading in place of thermal loading. Undoubtedly, it is a fast, simple, and convenient approach to rank the relative cracking performance of asphalt mixtures under a set of standard conditions. Furthermore, it can also simulate mechanical loading associated with cracking phenomena, such as surface-initiated longitudinal wheel path cracks which are usually caused by the higher tire pressures of truck wheels [38]. However, these laboratory tests cannot reflect some important factors that could lead to the observed cracking in the field, such as cooling rate [39], glass transition temperature (Tg) [40], thermal contraction coefficient [41], and reversible aging [42]. In addition, compared with mechanical load-induced local cracking, thermally induced shrinkage stress may result in more serious transverse cracking. Field monitoring and comparison of thermal- and mechanical-load-induced strains in asphalt pavement showed that although the frequency of thermal-induced strain was lower, resulting damage caused by thermal-induced strains could be more than that of mechanical load-induced strains, due to its higher amplitude [43]. From the perspective of prevention, thermally induced loading is hardly affected by human factors compared with mechanical-induced loading, which can be easily controlled by limiting the wheel load of trucks. Therefore, the asphalt thermal cracking analyzer (ATCA) test [44], uniaxial thermal stress and strain test (UTSST) [45], and asphalt concrete cracking device (ACCD) test [37] were developed for the optimized selection of materials with improved resistance to thermal shrinkage cracking. Since cracking propagation is an important property for understanding the cracking mechanism of pavements, Mandal and Bahia [46] also proposed to test notched samples in a restrained configuration.

## 3. FE Model Development

### 3.1. Geometry and Structure of Model

**Figure 1:**
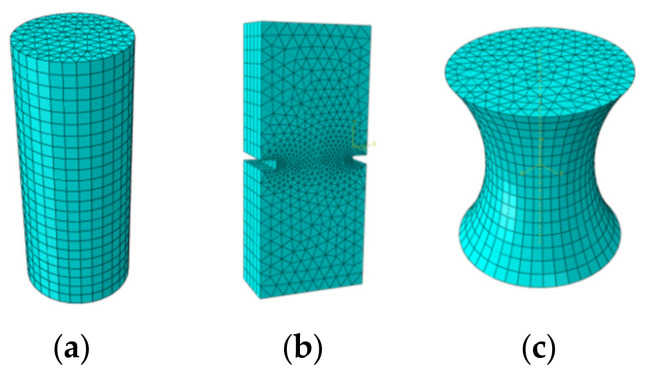
Three-dimensional FE model of selected sample geometries after meshing. (**a**) Cylindrical geometry; (**b**) double-notched geometry; and (**c**) dog-bone geometry.

In order to evaluate the influence of sample geometry, two kinds of load, mechanical and thermally induced loads, were applied on the finite element model (FEM) of samples with different geometries using finite element simulation software ABAQUS^®^ ver.6.12 [47]. The boundary conditions played a major role in predicting the response of the model. Consequently, fixed boundary conditions at top and bottom surfaces, and fixed boundary conditions only at the bottom surface were applied for thermal load samples and mechanical load samples, respectively. Due to the importance of the meshing step of the model in obtaining the most accurate results, many trials were undertaken during simulation to determine the best mesh size. The six-node wedge element (C3D6) was utilized to mesh the model, as shown in Figure 1. Through setting a correct mesh size and step analysis, a simulation with higher accuracy and smaller computational time can be obtained.

### 3.2. Material Characterization

In this study, asphalt mixture type was a dense-graded asphalt concrete (AC-20) with nominal maximum aggregate size (NMAS) of 20 mm which was characterized as a linear viscoelastic material. Hooke’s law was utilized to describe the behavior of linear elastic material. The elastic properties of the asphalt material are given in Table 3.

The Burgers model is usually employed by researchers to describe the viscoelastic properties of asphalt mixture. However, the Burgers model can only reflect the deformation characteristics of asphalt mixture in the short term. Thus, the generalized Maxwell model was developed by assembling several Maxwell models in parallel to better describe the relaxation performance of asphalt mixture in the long term [50]. The relaxation model adopted in ABAQUS^®^ finite element software is the Prony model, which has the same mathematical expression as the generalized Maxwell model. A 9-element Prony’s series was used to define the relaxation modulus at the reference temperature of 20 °C. The Prony’s series model parameters are shown in Table 4. The Williams–Landel–Ferry (WLF) equation parameters were obtained from conducting dynamic modulus tests on the same asphalt materials at the same reference temperature and are presented in Table 5 [49].

In order to consider the temperature field transmission process from outside to the central section of the model, the coefficients of thermal contraction at different temperatures are provided in Table 6. The values of the thermal properties of asphalt mixture were acquired from previous works in the literature [51]. The thermal conductivity and the specific heat of the asphalt mixture used in this study were 0.74 W/(m∙°C) and 880 J/(kg∙°C), respectively.

## 4. Results and Discussion

### 4.1. Effects of Sample Geometry on Stress State in Asphalt Mixture

**Figure 2:**
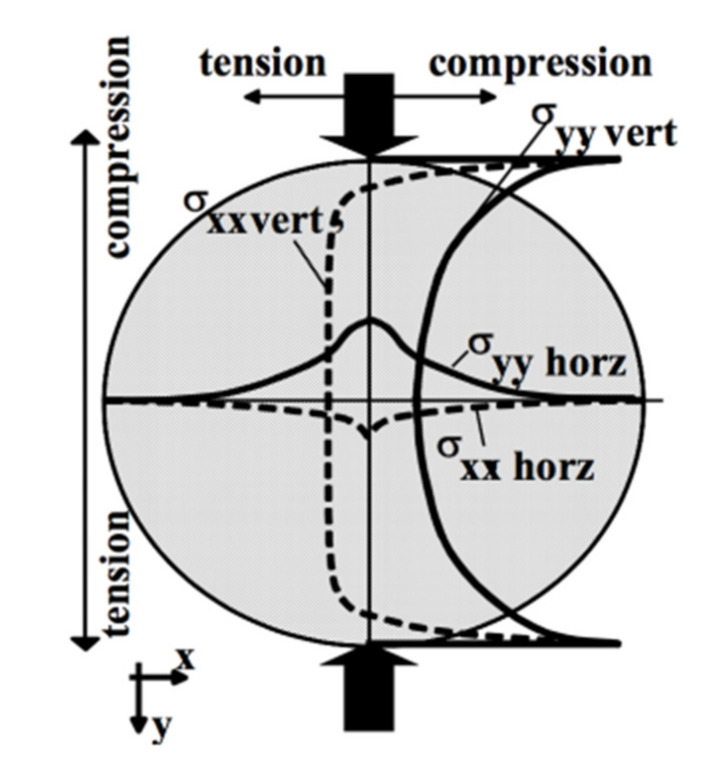
Stress distribution along the vertical and horizontal directions using indirect tensile test.

Indirect tensile test configuration is widely utilized to evaluate the cracking resistance of asphalt mixture because it is simple to carry out without requiring cumbersome cutting or gluing procedures. Using theoretical mechanics, it is straightforward to obtain the stress distribution in testing specimens under mechanical loading. Figure 2 shows the stress distribution along the vertical and horizontal directions of the asphalt mixture sample. It can be seen that the internal stress distribution is complex, and the values obtained from the test are not “real” tension values. If the horizontal and vertical strains at the center of the specimen are measured, a reliable Poisson’s ratio can be determined. The stiffness values are always rough approximations, because there exists no direct relationship between the applied force and the stress at the center as a result of the complicated internal stress distribution [52]. Therefore, the observed response is related to a particular state of stress and cannot be generalized in other situations.

**Figure 3:**
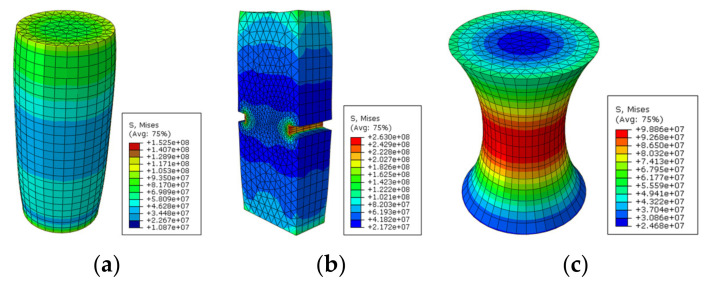
Stress state in direct tensile test configurations. (**a**) Cylindrical geometry; (**b**) double-notched geometry; and (**c**) dog-bone geometry.

Compared with direct tensile configuration, compressive stress clearly exists in other common test configurations such as three-point bending, single-edge notched beam, semi-circular bending/tensile, and disk-shaped compact tensile tests. It is well recognized that asphalt pavement cracking performance in the field mainly depends on the tensile properties of asphalt binders. The ideal stress state in a tested sample is a simple tensile. Except for the binder, there are also many factors which would affect the compression behavior of the asphalt mixture, such as aggregate gradation, aggregate strength, and interlock effects. Therefore, the final tensile failure strain would be affected by the abovementioned factors, which are not consistent with the real field conditions. Based on this analysis, only three direct tensile test configurations were considered for numerical simulation analysis, as shown in Figure 3. It is evident that an almost uniform stress state existed for the cylindrical geometry. Moreover, there was an obvious stress concentration in both the notched sample and the dog-bone sample. The advantages of this stress concentration are that, firstly, the failure plane is known in advance, and secondly, it is possible to install gauges at the failure plane for measuring deformation-related properties.

**Figure 4:**
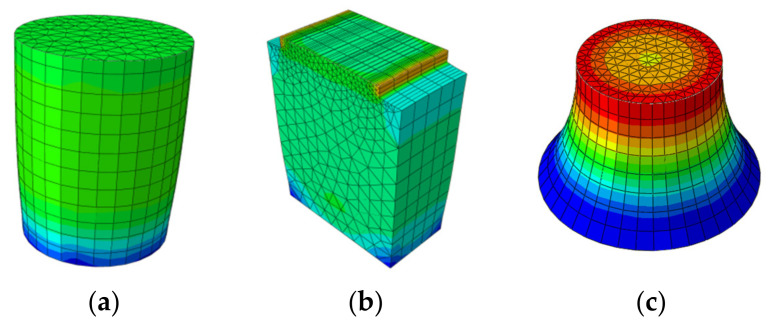
The stress states of middle cross sections: (**a**) cylindrical geometry; (**b**) double-notched geometry; and (**c**) dog-bone geometry.

The middle cross section of the stress states for the three configurations are shown in Figure 4. It is evident that stress on the edge of the notched sample was significantly higher than the stress in the middle part, thereby suggesting that the crack propagation in notched geometry is straightforward. It is a controversial topic as to whether a notched asphalt mixture sample should be utilized for testing or not. Some researchers hold the opinion that tests performed on a notched sample can better differentiate materials with different compositions. In addition, it is believed that it would be beneficial to use a notched specimen when measuring the temperature at which the mixture becomes vulnerable to cracking [53]. However, it should be noted that the notches have an extremely high possibility of passing through the aggregates, owing to the high proportion of aggregates in the mixture, and that is not consistent with what occurs in the field. Furthermore, it is difficult to control the crack initiation, which could lead to uncertainty in the test.

### 4.2. Comparison between Thermal and Mechanical Load Modes

**Figure 5:**
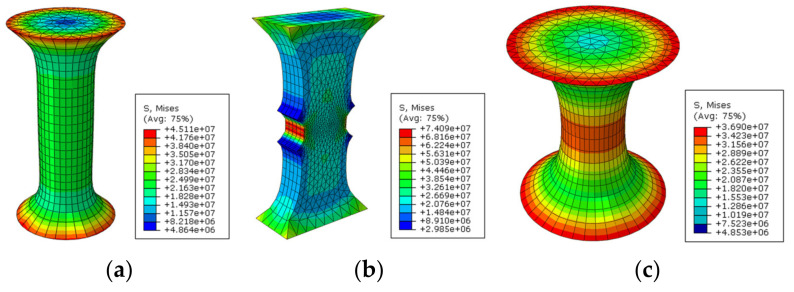
Effects of thermal load on asphalt mixture response direct tensile test configurations. (**a**) Cylindrical geometry; (**b**) double-notched geometry; and (**c**) dog-bone geometry.

In order to realize the difference between the influence of the thermal load and the mechanical load on mixture response, thermal load was also applied to asphalt mixtures with three different direct tensile configurations. The numerical results are shown in Figure 5. It can obviously be seen that thermal load had a symmetrical effect on the mechanical response. For the notched sample, it is certain that the failure plane appeared in the middle cross section due to a higher degree of stress concentration. However, local stress concentration appeared at both ends for the other two geometries, resulting in localized damage and fracture near the loading platens. This phenomenon can be attributed to the fully constrained boundary conditions. One special treatment could be the application of the glue at both ends of the sample before performing the test. Similar to mechanical loading, the stress around the specimen was higher than that at the core of the sample. Some researchers applied a compressive mechanical loading with the rate of 1.27 mm/min in indirect tensile test (IDT) to represent thermally induced tensile loading rate [54]. However, if it is assumed that the temperature decreases at a rate of 10 °C per hour, as usually applied in TSRST, and a typical contraction coefficient for asphalt mixture is approximately taken as 2 × 10^−5^ per degree, it will result in a loading rate of 0.001 mm/min. That is three orders of magnitude less than the mechanical load, with a loading rate of 1.27 mm/min. Different loading rates may change the failure mode in the asphalt mixture, e.g., a higher loading rate may readily cause the fracture of coarse aggregate [36].

## 5. Conclusions

This numerical study aimed to investigate the effects of geometry and loading mode on the stress state of asphalt mixture cracking tests. Considering the results presented in this paper, the following conclusions can be obtained:

(1) The application of thermally induced load in a restrained uniaxial test configuration should be considered when asphalt mixture cracking characterization is carried out. Consequently, important factors, such as cooling rate, glass transition temperature, thermal contraction coefficient, and reversible aging, which influence the observed cracking in the field, are of great importance during testing;
(2) Compared with direct tensile configuration, compressive stress clearly exists in other common test configurations, such as three-point bending, single-edge notched beam, semi-circular bending/tensile, and disk-shaped compact tensile tests, which may prevent the initiation and propagation of cracks;
(3) Although the test on a notched sample can better characterize the cracking propagation process, it should be noted that notches have an extremely high possibility of passing through the aggregates due to their high proportion in the mixture, and that is not consistent with what happens in the field. In addition, it is difficult to control the crack initiation which can lead to uncertainty in the test;
(4) A uniform stress state almost exists in cylindrical geometry. Moreover, there is an obvious stress concentration for notched and dog-bone samples. The advantages of this stress concentration are that, firstly, failure plane is known in advance, and secondly, it is possible to install gauges at the failure plane for measuring deformation-related properties.

## 6. Future Work

The future studies on this research topic could be continued in the following two directions:

(1) In order to simplify the analysis of numerical simulation, asphalt mixture, in this study, was considered as a single-phase material with a single constitutive relationship. In fact, at least two phase materials (binder and coarse aggregate) exist in the mixture. For thinner asphalt mixture samples or a notched sample, size effects should not be ignored. Furthermore, aggregate and binder or mastic have totally different thermal contraction coefficients and moduli. Thus, micromechanical analysis of thermal stresses and strains with multi-phase models from the image scanning of a sample cross section is imperative;
(2) Laboratory restrained notched cooling tests could be performed on asphalt mixtures containing asphalt binders with totally different reversible aging trends to observe if reversible aging in asphalt binder will transfer into the properties of asphalt mixture. Since laboratory asphalt mixture aging procedures may not fully consider the field condition [55,56], more harsh asphalt mixture aging protocol should be considered before running cracking tests.

## Figures and Tables

**Table 1:** Specimen geometry and features of common unnotched asphalt mixture fracture test configurations (tests on unnotched samples).

Specimen Geometries	Features
Indirect tensile [26,27] 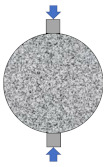	Only applicable for mechanical loading. Extremely easy to perform. The internal stress distribution is complex. Not “real” tension values obtained.
Mixture BBR [28] 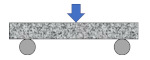	Only applicable for mechanical loading. Small thickness of beam allows analysis of the effect of aging at very small pavement depths. Binder properties can be back-calculated using appropriate composite material model. The volume of material tested may not be representative.
Four-point bending [29] 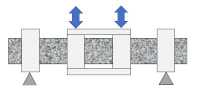	Only applicable for cyclic mechanical loading. Cyclic load needs longer time. Results depend on a combination of structural and materials properties.
Overlay tensile [29] 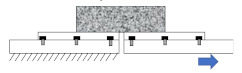	Only applicable for mechanical loading. A structural test to simulate reflective cracking. No fundamental material property is related
Restrained cooling [30] 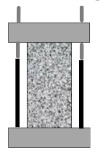	Applicable for mechanical and thermal loading. Possibility of load eccentricity due to end fixtures. Misalignment may cause bending. The position of specimen failure is unknown and in case of failure at the caps the stress distribution is unknown.
Dog-bone shape direct tensile [30] 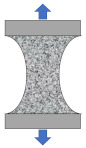	Applicable for mechanical and thermal loading. Subjected to a known and simple state of stress. Misalignment may cause bending. Sample failures at a known position.
Hollow cylinder tensile [30] 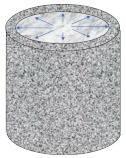	Only applicable for mechanical loading. Complex special devices are needed. Can obtain fundamental material properties. No stress concentration at the point of load application.

**Table 2:** Specimen geometries and features of common notched asphalt mixture fracture test configurations (test on notched sample).

Specimen Geometry	Features
Single-edge notched beam [31] 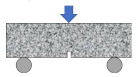	Constrained for crack propagation. Higher fracture surface area. Only applicable for mechanical loading.
Semi-circular bending [32] 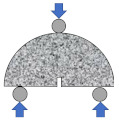	Complicated stress distribution. Constrained for crack propagation. Smaller fracture surface area. Only applicable for mechanical loading.
Semi-circular tensile [33] 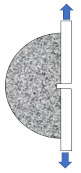	Applicable for mechanical and thermal loading. Generates tensile stresses around the cracking area. Cracking can easily propagate. Similar to semi-circular bending (SCB) test.
Disk-shaped compact tensile [34] 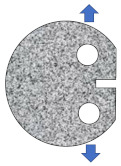	Complex stress distribution. Failure around the loading holes. Larger fracture surface area. Applicable for mechanical and thermal loading.
Indirect ring tensile [35] 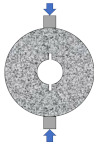	Higher fracture surface area. Only applicable for mechanical loading. Similar test frame as indirect tensile test (IDT). Pure tensile state along the crack propagation line.
Double-edge notched tensile [36] 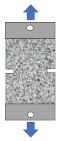	Applicable for mechanical and thermal loading. Pure tension state along the crack propagation line. Results can be analyzed using essential work of fracture.
Restrained notched ring [37] 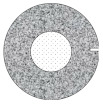	Only applicable for thermal loading. Sample compaction needs special equipment. Directly measure the cracking resistance under field-like conditions.

**Table 3:** Elastic material properties [48,49].

Material	Moduli (MPa)	Poisson’s Ratio	Density (kg/m^3^)
AC-20	14,500	0.35	2400

**Table 4:** Prony series model parameters (*E*_0_ = 14,500 MPa).

N (Number of Maxwell Units)	G (Ratio of Normal Modulus)	K (Ratio of Tangent Modulus)	*τ* (Relaxation Time)
1	0.1679	0	0.00001
2	0.1793	0	0.0001
3	0.2555	0	0.001
4	0.2194	0	0.01
5	0.1049	0	0.1
6	0.0368	0	1
7	0.0129	0	10
8	0.0048	0	100
9	0.0022	0	1000

**Table 5:** WLF equation constants of asphalt materials [49].

Mixture Type	C_1_	C_2_
AC-20	33.5	284.9

**Table 6:** Thermal contraction and expansion coefficients.

N	Contraction and Expansion Coefficients	Temperature/°C
1	1.00 × 10^−5^	40
2	1.20 × 10^−5^	30
3	1.50 × 10^−5^	25
4	1.80 × 10^−5^	20
5	2.10 × 10^−5^	15
6	2.40 × 10^−5^	10
7	2.60 × 10^−5^	0
8	2.10 × 10^−5^	−10
9	1.60 × 10^−5^	−20
10	1.30 × 10^−5^	−26

## Data Availability

Not applicable.
